# Metabolic and inflammatory traits and depression: a Mendelian randomization study with late-adolescent population-based and clinical triangulation

**DOI:** 10.3389/fpsyt.2026.1935185

**Published:** 2026-10-16

**Authors:** Yanping Wu, Zhenyu Xu, Daolin Wu

**Affiliations:** Department of Psychiatry, Ganzhou Hospital–Nanfang Hospital, Southern Medical University (Ganzhou People’s Hospital), Ganzhou, Jiangxi, China

**Keywords:** adolescent, C-reactive protein, depressive disorder, insulin resistance, Mendelian randomization analysis, triglyceride-glucose index

## Abstract

**Objective:**

Metabolic dysfunction and systemic inflammation have been associated with adolescent depression, but their causal relevance remains uncertain. We examined whether insulin resistance, proxied by the triglyceride-glucose (TyG) index, and high-sensitivity C-reactive protein (hs-CRP) show genetically proxied associations with major depressive disorder (MDD), and evaluated their observational relevance in late adolescents from the general population and in a clinical adolescent sample.

**Methods:**

We performed bidirectional two-sample Mendelian randomization (MR) using genome-wide association study (GWAS) summary statistics for the TyG index, hs-CRP, body mass index (BMI), and MDD, with inverse-variance weighting as the primary method, supplemented by sensitivity and multivariable MR analyses. Observational corroboration was sought in 327 late adolescents aged 18–19 years from the pooled 2015–2016 and 2017–March 2020 pre-pandemic cycles of the National Health and Nutrition Examination Survey (NHANES) and in a retrospective cohort of 224 adolescents with depression and 200 frequency-matched controls.

**Results:**

Genetically predicted higher TyG index (odds ratio [OR] 1.08, 95% confidence interval [CI] 1.03–1.14, P = 0.001) and hs-CRP (OR 1.11, 95% CI 1.05–1.16, P<0.001) were associated with higher odds of MDD; reverse MR analyses did not show statistically significant associations. In the late-adolescent NHANES sample, each 1-SD higher TyG index (adjusted OR 1.28, 95% CI 1.01–1.63) and each 1-SD higher log-transformed hs-CRP (adjusted OR 1.33, 95% CI 1.03–1.71) were associated with higher odds of moderate-to-severe depressive symptoms (Patient Health Questionnaire-9 score ≥10). In the clinical cohort, the TyG index (adjusted OR 1.35, 95% CI 1.15–1.58) and hs-CRP >3.0 mg/L (adjusted OR 2.55, 95% CI 1.60–4.06) were associated with higher odds of depression. In a multiple-imputation sensitivity analysis retaining all 561 exposure/outcome-complete NHANES participants, both NHANES associations remained directionally consistent with the primary complete-case estimates.

**Conclusion:**

Converging but methodologically distinct lines of evidence—genetic, population-based, and clinical—are directionally consistent with a contribution of insulin resistance and systemic inflammation to depression across adolescence and late adolescence. These findings support further prospective and interventional evaluation of metabolic-inflammatory pathways rather than immediate clinical translation.

## Introduction

Adolescence is a critical developmental period characterized by profound neurobiological, psychological, and social transformations (1). However, it is also a stage of heightened vulnerability to the onset of mental health disorders, with major depressive disorder (MDD) representing one of the most pressing public health challenges. Globally, depression is a leading cause of illness and disability among adolescents, with prevalence rates escalating in recent years (2). The consequences of adolescent depression are severe and far-reaching, extending beyond immediate emotional distress. It significantly impairs academic performance, strains peer and family relationships (3, 4), and tragically, is a primary risk factor for suicidal ideation and attempts (5). The long-term sequelae are equally concerning; individuals who experience depression during their teenage years are at a substantially higher risk for recurrent depressive episodes, anxiety disorders, substance abuse, and poor physical health outcomes throughout their lifespan (6). The societal and economic burden is immense, encompassing direct healthcare costs and indirect costs related to lost educational and occupational productivity (7). Despite the clear and growing impact of this condition, our understanding of its underlying pathophysiology remains incomplete, hindering the development of more effective, targeted prevention and intervention strategies. The traditional monoamine-based hypothesis of depression fails to fully explain its complex etiology, prompting a critical need to explore alternative or complementary biological pathways (8).

Emerging evidence points towards a compelling link between systemic physiological processes and mental health, with metabolic dysregulation and chronic low-grade inflammation standing out as two interconnected and highly plausible contributors to depression (9). Metabolism and inflammation are intrinsically linked in a bidirectional relationship; metabolic disturbances, such as insulin resistance and obesity, can trigger and sustain a state of chronic inflammation by activating innate immune pathways (10). Adipose tissue, for instance, is not merely a passive energy reservoir but an active endocrine organ that releases pro-inflammatory mediators such as interleukin-6 (IL-6) and tumor necrosis factor-alpha (TNF-α) and can thereby contribute to increased hepatic C-reactive protein (CRP) production (11, 12). Conversely, chronic inflammation can disrupt metabolic homeostasis, for example, by impairing insulin signaling, thereby exacerbating insulin resistance (10). Both pathways have been independently implicated in the pathophysiology of depression. Metabolic abnormalities may affect brain function through mechanisms like impaired cerebral glucose utilization, altered neuroplasticity, and hypothalamic-pituitary-adrenal (HPA) axis dysfunction (13, 14). Simultaneously, the “inflammatory hypothesis of depression” posits that elevated peripheral cytokines can cross the blood-brain barrier, activating microglia and leading to neuroinflammation, which in turn disrupts neurotransmitter synthesis, reduces neurotrophic support, and ultimately contributes to the development of depressive symptoms (8). Preclinical studies in adolescent models suggest that inflammatory, oxidative, and neurochemical processes can converge on depressive-like behavior (15, 16). However, integrated human evidence on metabolic-inflammatory dysregulation remains limited.

To date, the investigation into the tripartite relationship between metabolism, inflammation, and depression has been dominated by observational studies. Such studies have linked metabolic dysfunction and inflammatory markers with depression or depressive symptoms across population-based and youth studies (17, 18). However, they are fraught with methodological limitations that preclude causal inference. A primary issue is the potential for confounding; factors such as diet, physical activity, sleep patterns, and socioeconomic status are intricately linked to all three domains and may distort the observed associations (19, 20). Furthermore, the directionality of the relationship is ambiguous due to the risk of reverse causation, where depression itself may lead to lifestyle changes that promote metabolic and inflammatory disturbances (21, 22). Adolescent-specific biomarker evidence remains comparatively limited and heterogeneous, with studies often focusing on individual inflammatory markers or biomarker panels rather than integrated metabolic-inflammatory pathways (23, 24). Genetically informed methods such as Mendelian randomization (MR) can help dissect causality, although the available genome-wide association study (GWAS) resources for metabolic and inflammatory traits are themselves derived predominantly from adult or mixed-age populations of European ancestry and therefore cannot, on their own, establish effects specific to adolescent-onset depression. Without establishing a causal link, it remains uncertain whether targeting metabolic and inflammatory pathways would be an effective strategy for preventing or treating depression (25, 26).

Therefore, the present study was designed to address these critical gaps by employing a multi-faceted approach to rigorously investigate the putative etiologic relationship between metabolic-inflammatory dysregulation and depression. Our primary aim was to utilize a two-sample Mendelian randomization (MR) design (27), leveraging large-scale genetic data to estimate genetically proxied associations of insulin resistance (proxied by the TyG index) and systemic inflammation (proxied by hs-CRP) with MDD; we interpret these MR estimates as reflecting genetic liability across the life course rather than effects specific to adolescence. The key advantage of MR is its use of randomly allocated genetic variants as instrumental variables, which are less susceptible to confounding and reverse causation than conventional observational designs (28). To triangulate our findings and enhance their clinical relevance (29), we further aimed to evaluate these associations observationally in two distinct settings: first, in a nationally representative sample of US late adolescents aged 18–19 years from the NHANES database, and second, in a retrospective clinical cohort of adolescents aged 12–19 years from our own clinical practice. Because the three components differ in age structure, phenotype ascertainment, and susceptibility to confounding, we interpret them within a triangulation framework (29) rather than as mutual validation. By integrating evidence from genetic, population, and clinical data, this study seeks to provide a comprehensive and robust assessment of the role of metabolic-inflammatory pathways in the etiology of depression during adolescence.

## Materials and methods

### Study design

This investigation employed a three-stage triangulation design (29) combining genetically informed and observational evidence; each component was interpreted within its own evidentiary limits rather than as a validation hierarchy. The specific data sources, inclusion/exclusion criteria, and sample selection flow for each stage are detailed in **Supplementary Table S0**. First, we conducted a bidirectional two-sample Mendelian randomization (MR) analysis to estimate genetically proxied associations using large-scale GWAS summary statistics. Second, we performed a cross-sectional corroborative analysis using nationally representative data from the U.S. National Health and Nutrition Examination Survey (NHANES), restricted to late adolescents aged 18–19 years (final analytic N = 327). Third, we conducted a retrospective case-control study using clinical data from our institution to examine the same associations in a real-world adolescent patient population. This report was prepared with reference to the STROBE-MR (Strengthening the Reporting of Observational Studies in Epidemiology using Mendelian Randomization) recommendations (30); a completed STROBE-MR checklist is provided as a **Supplementary File**. An overview of the study design is shown in **Figure 1**.

**Figure 1:**
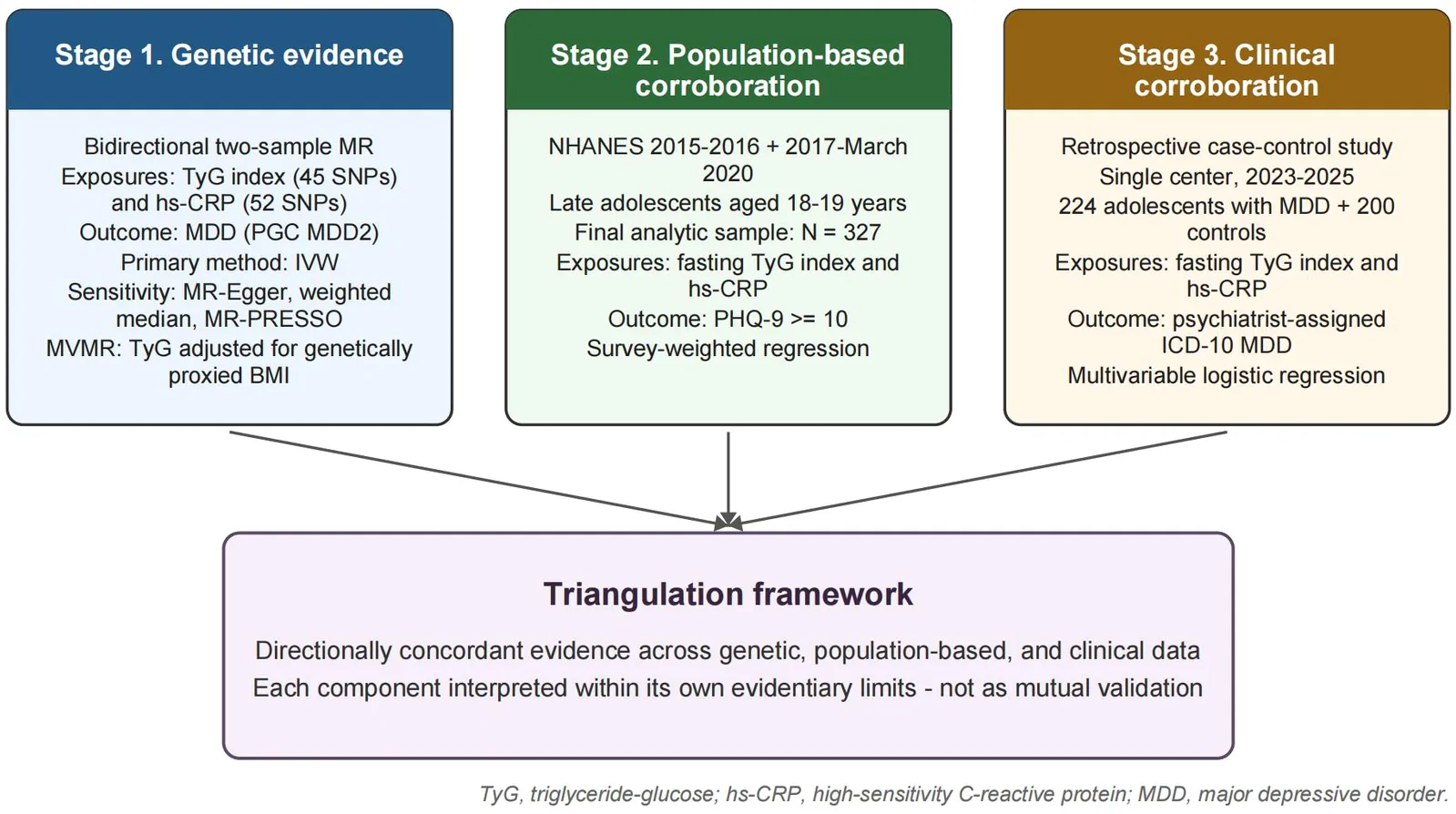
Overview of the three-stage triangulation design. GWAS, genome-wide association study; IVW, inverse-variance weighted; MVMR, multivariable Mendelian randomization; NHANES, national health and nutrition examination survey; PHQ-9, patient health questionnaire-9; MDD, major depressive disorder; EMR, electronic medical records.

The study was conducted in accordance with the principles of the Declaration of Helsinki. The protocol for the retrospective clinical cohort study was exempted from formal ethical review by the Institutional Review Board (IRB) of Ganzhou Hospital–Nanfang Hospital, Southern Medical University (Ganzhou People’s Hospital) (Exemption Number: GZPH-IRB-2024-001). For the publicly available NHANES dataset and GWAS summary statistics, ethical approval and informed consent were previously obtained by the original investigators, and the use of these datasets in the current study was exempted from further ethical review. The MR design relies on three core instrumental-variable assumptions: (1) relevance, whereby the genetic variants are robustly associated with the exposure; (2) independence, whereby the variants are independent of confounders of the exposure–outcome association; and (3) exclusion restriction, whereby the variants influence the outcome only through the exposure.

### Data sources and study population

1. Mendelian Randomization (Genetic Analysis): Summary-level statistics were obtained from large-scale GWAS conducted predominantly in adult or mixed-age populations of European ancestry; detailed characteristics and dataset/resource identifiers are provided in **Supplementary Table S1**. Genetic instruments for the TyG index were derived from a UK Biobank–based GWAS of 273,368 European-ancestry participants aged 40–69 years who were free of diabetes mellitus and disorders of lipoprotein metabolism; the source study calculated the TyG index from random (non-fasting) plasma triglyceride and glucose measurements (31). Instruments for hs-CRP were derived from the UK Biobank biomarker GWAS of 363,228 individuals of predominantly European ancestry (32). Instruments for BMI (used in the multivariable MR) were obtained from the GIANT + UK Biobank meta-analysis of approximately 681,275 European-ancestry individuals (33). Summary-level data for the outcome, MDD, were obtained from the Psychiatric Genomics Consortium (PGC) MDD2 meta-analysis (135,458 cases and 344,901 controls), in which case definitions combined structured diagnostic interviews, clinician diagnoses, and electronic health record– or self-report–based phenotypes across contributing cohorts (34). Because the PGC MDD2 meta-analysis included a UK Biobank cohort, overlap with the UK Biobank–based TyG and hs-CRP exposure GWAS was possible and could not be quantified precisely from the summary files used here; strong instruments reduce, but do not eliminate, the potential for overlap-related bias (35). Differences in population structure across contributing cohorts (e.g., UK Biobank versus other European-ancestry PGC cohorts) may additionally leave residual population stratification, which we consider when interpreting the MR estimates. No FinnGen summary statistics were used in the reported MR analyses.

2. NHANES Observational Analysis: We pooled publicly available data from NHANES 2015–2016 and the 2017–March 2020 pre-pandemic release. Because publicly released PHQ-9 data in these cycles are available only for participants aged 18 years or older (data for participants aged 12–17 years are restricted-use through the NCHS Research Data Center), the present analysis was restricted to late adolescents aged 18–19 years (36). After applying the age criterion and excluding participants with incomplete PHQ-9, fasting triglyceride, fasting glucose, hs-CRP, or required covariate data, and after applying the hs-CRP >10 mg/L exclusion rule for the primary analysis (see below), 327 participants were included in the final analytic sample (**Figure 2**). The initial age-eligible records numbered more than 2,000; stepwise exclusion counts were exported from the analysis pipeline and are shown in **Figure 2**. Covariate-specific missingness was quantified among the 561 participants with complete exposure and outcome data who met the primary hs-CRP criterion, before complete-case selection (see Statistical Analysis and **Supplementary Table S7**).

**Figure 2:**
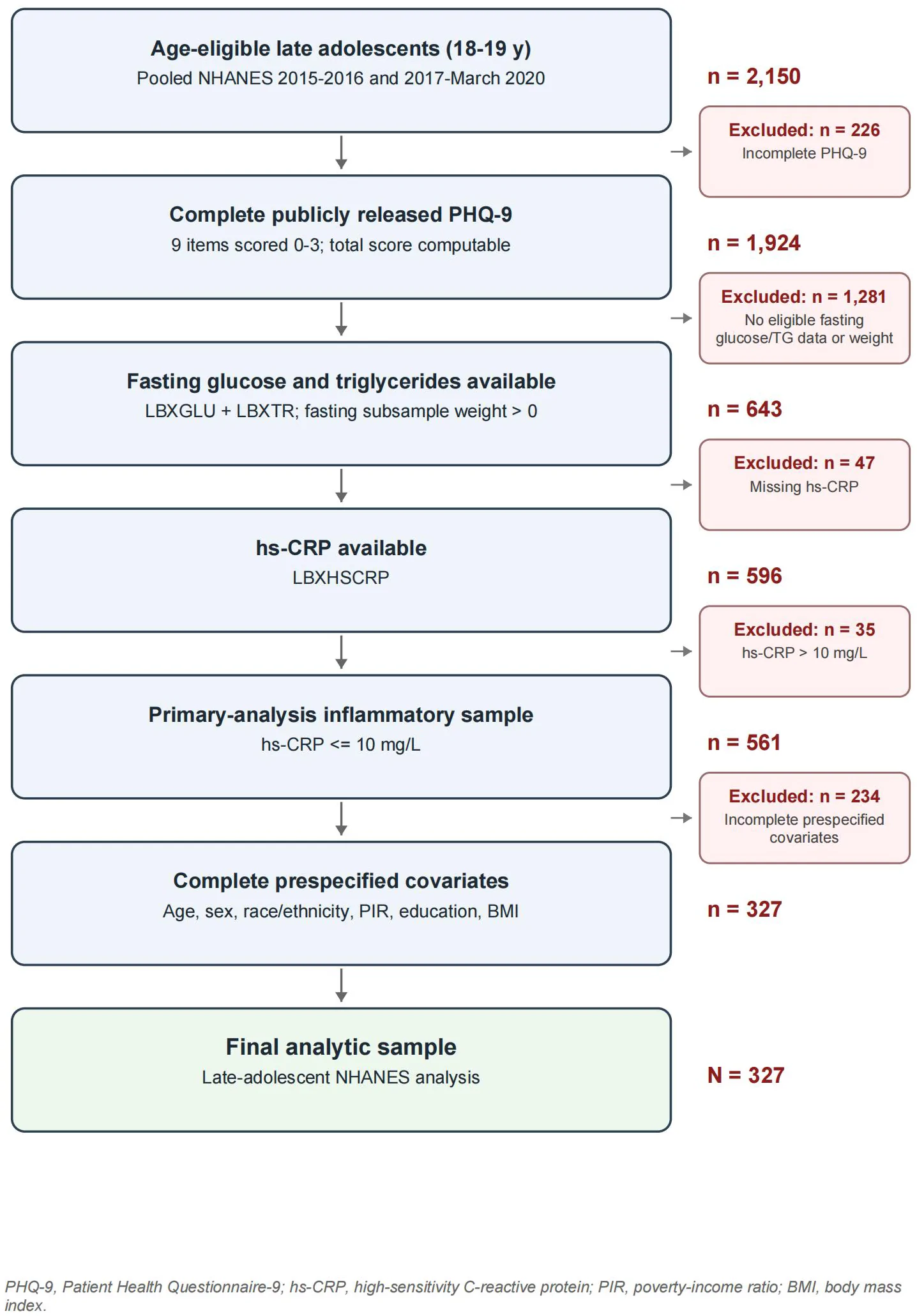
Flow diagram of the NHANES analytic sample (pooled 2015–2016 and 2017–March 2020 pre-pandemic cycles; late adolescents aged 18–19 years). hs-CRP, high-sensitivity C-reactive protein; PIR, poverty-income ratio; BMI, body mass index. Missing-covariate sensitivity analyses using multiple imputation retained all 561 exposure/outcome-complete participants.

Depressive symptoms were assessed with the nine-item Patient Health Questionnaire-9 (PHQ-9), a self-report measure of depressive symptom frequency over the preceding two weeks. Each item is scored on its original 0–3 Likert-type scale (“not at all” to “nearly every day”; items were not dichotomized), yielding a total score of 0–27; the questionnaire was administered by trained NHANES interviewers in the Mobile Examination Center using computer-assisted personal interviewing (CAPI), with standardized instructions provided to participants (36). A total score ≥10 was prespecified to define moderate-to-severe depressive symptoms, consistent with the original validation study (37) and with evidence supporting the validity of the PHQ-9 in adolescent populations (38); internal consistency in our analytic sample was acceptable to good (Cronbach’s α = 0.87). The TyG index was calculated as ln[fasting triglycerides (mg/dL; variable LBXTR) × fasting glucose (mg/dL; variable LBXGLU)/2]; all lipid and glucose measurements used fasting subsample specimens. Serum hs-CRP (variable LBXHSCRP) was measured with high-sensitivity assays in both cycles—using the Beckman Coulter Synchron platform in 2015–2016 and a two-reagent immunoturbidimetric method in 2017–March 2020—according to NCHS laboratory documentation (39). Socioeconomic position was indexed by the family poverty-income ratio (PIR; variable INDFMPIR), computed by NCHS as the ratio of family income to the federal poverty threshold specific to family size and composition, and was analyzed both continuously and in categories (<1.3, 1.3–3.5, >3.5). Educational attainment (variable DMDEDUC3, available for ages 6–19 years) was additionally recorded and collapsed into “less than high school” versus “high school or above” for sensitivity analyses. Following the CDC/AHA scientific statement, hs-CRP values >3.0 mg/L were used as a secondary categorical threshold for elevated inflammation (40); this threshold is descriptive and cardiometabolic in origin rather than an adolescent-specific depression cutoff, although it has also been applied in national analyses of US adolescents, among whom 15.3% overall (19.1% of those aged 16–19 years) exceeded it (41). For the primary analysis, participants with hs-CRP >10 mg/L were excluded to reduce the influence of acute infection or acute-phase responses, recognizing that this cutoff does not perfectly discriminate acute from chronic inflammation (42); sensitivity analyses including these participants were performed (see Statistical Analysis).

3. Retrospective Clinical Cohort: We recruited a retrospective cohort from the Electronic Medical Records (EMR) system of our hospital between July 2023 and July 2025. Case Group: Included 224 adolescents (aged 12–19 years) formally diagnosed with MDD according to ICD-10 criteria (codes F32.x or F33.x) by board-certified psychiatrists. Control Group: Included 200 age- and sex-matched adolescents who underwent routine health examinations during the same period and had no history of psychiatric disorders. Matching was performed at the group level (frequency matching on age and sex strata) rather than by individual 1:1 pairing; accordingly, unconditional logistic regression with adjustment for the matching factors was used (see Statistical Analysis). For all case participants, fasting venous blood (≥8 h fasting, morning draw) used for the present analyses was collected at the diagnostic work-up visit before initiation of psychotropic medication for the current depressive episode; serum triglycerides, fasting glucose, and hs-CRP were measured on the hospital’s clinical chemistry platform using an immunoturbidimetric hs-CRP assay (within- and between-run coefficients of variation <5% and <10%, respectively). Eleven case participants (4.9%) had a documented history of psychotropic medication exposure during an earlier treatment period; all had discontinued psychotropic medication for at least 12 months before the index blood draw. Sensitivity analyses excluding these participants were performed. Individual-level socioeconomic variables (e.g., family income or parental education) were not systematically recorded in the EMR and were therefore unavailable for adjustment. Exclusion Criteria: To ensure the specificity of metabolic and inflammatory markers, we excluded participants with: (1) active acute infections (e.g., hs-CRP > 10 mg/L), consistent with recommendations intended to reduce misclassification from acute-phase responses (42); (2) autoimmune diseases; (3) type 1 diabetes; or (4) current use of systemic corticosteroids, antibiotics, or lipid-lowering medications.

### Instrumental variable selection

For the MR analysis, genetic instruments (single nucleotide polymorphisms, SNPs) were selected based on three core assumptions. We selected independent SNPs (LD clumping threshold r2<0.001 within a 10,000 kb window) that were strongly associated with the exposure traits at a genome-wide significance level (P<5×10−8). The strength of the selected instruments was confirmed by an F-statistic > 10 to minimize weak instrument bias. Per-SNP F-statistics and the proportion of exposure variance explained (R² = 2 × EAF × (1 − EAF) × β², where EAF is the effect allele frequency and β the exposure association) were computed from the GWAS summary statistics. No proxy SNPs were used; variants unavailable in the outcome dataset were excluded after harmonization. Palindromic SNPs with intermediate effect allele frequencies (minor allele frequency >0.42) were excluded to avoid strand ambiguity. The MR analysis comprised two forward analyses (TyG index → MDD; hs-CRP → MDD) and two reverse analyses (MDD → TyG index; MDD → hs-CRP). BMI was not treated as a primary exposure in the bidirectional analysis and entered only the multivariable MR (MVMR) as a covariate. The full list of instrument SNPs, including effect and other alleles, effect sizes, standard errors, EAF, per-SNP R² and F-statistics, and harmonization status, is provided in **Supplementary Data File S1**; aggregate instrument-strength metrics are summarized in **Supplementary Table S2**.

### Data collection and variables

In the NHANES analysis, the study population consisted of 327 late adolescents aged 18–19 years. The primary outcome was moderate-to-severe depressive symptoms, defined as a PHQ-9 score of 10 or higher; throughout this report, NHANES results are described as depressive symptoms rather than clinically diagnosed MDD. Exposure variables included the TyG index, calculated as ln[fasting triglycerides (mg/dL) × fasting glucose (mg/dL)/2], and hs-CRP, which was log-transformed for primary analyses because of its right-skewed distribution.

In the retrospective clinical cohort, we identified 224 pediatric patients (aged 12–19 years) diagnosed with depression and an age- and sex-matched control group of 200 adolescents without psychiatric diagnoses. Data on fasting lipid profiles (triglycerides), fasting glucose, and hs-CRP were extracted, and the TyG index was calculated accordingly.

### Statistical analysis

All statistical analyses were performed using R software (version 4.3.1; R Foundation for Statistical Computing, Vienna, Austria). For the MR analysis, we performed both forward and reverse analyses. The random-effects inverse-variance weighted (IVW) method was used as the primary approach to estimate the genetically proxied association under MR assumptions. This was supplemented with three main sensitivity analyses: MR-Egger regression to detect and adjust for directional pleiotropy, the weighted median method, which provides robust estimates even when up to 50% of instruments are invalid, and the MR-PRESSO test to identify and correct for pleiotropic outliers. Multivariable MR (MVMR) was performed to estimate the association of genetically proxied TyG index with MDD after accounting for genetically proxied BMI. MR analyses used the TwoSampleMR package (functions harmonise_data, mr, mr_heterogeneity, and mr_pleiotropy_test) and the MRPRESSO package (function mr_presso). The two primary forward IVW hypotheses (TyG → MDD and hs-CRP → MDD) were evaluated against a Bonferroni-adjusted threshold of P < 0.025; reverse MR and sensitivity estimators are reported with nominal P values, effect directions, and confidence intervals.

NHANES analyses accounted for the complex sampling design using masked strata (SDMVSTRA) and primary sampling units (SDMVPSU). Because the pooled dataset combined a 2-year cycle (2015–2016) with the 3.2-year 2017–March 2020 pre-pandemic release, fasting subsample weights were rescaled to represent the combined 5.2-year period in accordance with NCHS analytic guidance (43): the 2015–2016 fasting weight (WTSAF2YR) was multiplied by 2/5.2 and the 2017–March 2020 fasting weight (WTSAFPRP) by 3.2/5.2. Survey-weighted logistic regression was fitted with the survey package (functions svydesign and svyglm, with nest = TRUE). Baseline characteristics are reported as raw numbers with survey-weighted percentages. Primary exposures were analyzed continuously (TyG index per 1-SD; log-transformed hs-CRP per 1-SD). Three adjustment sets were used: Model A adjusted for age and sex; Model B additionally adjusted for race/ethnicity and PIR; Model C additionally adjusted for BMI. Categorical analyses (TyG tertiles; hs-CRP <1.0, 1.0–3.0, and >3.0–mg/L) were treated as secondary, and the TyG × hs-CRP multiplicative interaction was treated as exploratory. Sensitivity analyses comprised (i) continuous PHQ-9 score as the outcome; (ii) inclusion of participants with hs-CRP >10 mg/L; (iii) additional adjustment for educational attainment; and (iv) adjustment for survey period (2015–2016 vs 2017–March 2020). Given the modest number of outcome events, extensive subgroup analyses were not pursued. P values from secondary and exploratory NHANES analyses were adjusted using the Benjamini–Hochberg false discovery rate procedure (function p.adjust, method = “BH”); P values reported in earlier versions of this manuscript were nominal, and the correction approach is now stated explicitly.

The primary NHANES analysis retained the same fixed complete-case cohort (N = 327) used in the prior revision, requiring completeness for covariates used across the main and planned sensitivity models so that the reported estimates were based on a consistent analytic sample. To evaluate the potential impact of this approach, we quantified variable-specific missingness among the 561 participants with complete exposure and outcome data who met the primary hs-CRP criterion. We additionally compared participants retained in versus excluded from the complete-case sample using observed characteristics and standardized mean differences. As a sensitivity analysis, missing covariate values were imputed using multiple imputation by chained equations with 50 imputed datasets (44, 45). Only covariates were imputed—predictive mean matching for the poverty-income ratio and BMI, logistic regression for educational attainment, and polytomous regression for race/ethnicity—and the imputation model additionally included the exposures (TyG index, log hs-CRP), PHQ-9 outcomes, and survey period as predictors; TyG, hs-CRP, PHQ-9 outcomes, survey weights, strata, and primary sampling units were not imputed. Survey-weighted regression models were fitted within each imputed dataset using the same 5.2-year fasting subsample weights, and estimates were combined using Rubin’s rules.

For the clinical cohort analysis, covariates (age, sex, and BMI) were selected on the basis of prior clinical knowledge and the matching design rather than by univariate P-value screening, and were entered simultaneously into a multivariable logistic regression model (function glm, binomial family). Between-group comparisons of TyG index and hs-CRP levels used independent t-tests or Mann-Whitney U tests as appropriate. A two-sided P-value < 0.05 was considered statistically significant unless otherwise stated. Throughout, odds ratios from the cross-sectional and case-control analyses are interpreted as higher or lower odds, not as increases in absolute risk.

## Results

### Genetically proxied associations of metabolic and inflammatory traits with MDD

After a rigorous selection process, we identified 45 and 52 independent SNPs as instrumental variables for the TyG index and hs-CRP, respectively. All selected instruments were robust, exhibiting no weak instrument bias, with mean F-statistics of 419.2 for the TyG index and 581.5 for hs-CRP. The instrument sets explained 6.9% and 8.4% of the variance in the TyG index and hs-CRP, respectively (**Supplementary Table S2**); per-SNP characteristics, including effect sizes, allele frequencies, R², and F-statistics, are listed in **Supplementary Data File S1**.

In the primary forward MR analysis, genetically proxied higher levels of the metabolic-inflammatory markers were associated with higher odds of MDD. A genetically predicted 1-SD increase in the TyG index was associated with an 8% higher odds of MDD (IVW OR 1.08, 95% CI 1.03–1.14, P = 0.001), and a 1-SD increase in genetically predicted hs-CRP was associated with an 11% higher odds (IVW OR 1.11, 95% CI 1.05–1.16, P<0.001); both estimates survived the Bonferroni-adjusted threshold (P<0.025) but are modest in magnitude (**Table 1**; **Figure 3**). Effect directions were consistent across MR-Egger, weighted-median, and MR-PRESSO estimators. There was no strong evidence of heterogeneity (TyG: Cochran’s Q P = 0.18; hs-CRP: Q P = 0.09) or of directional pleiotropy (MR-Egger intercepts −0.002, P = 0.61, and 0.001, P = 0.72, respectively), and MR-PRESSO detected no significant outliers. In MVMR adjusting for genetically proxied BMI, the association of the TyG index with MDD persisted (OR 1.07, 95% CI 1.01–1.13), indicating an association independent of genetically proxied BMI; mutual adjustment of TyG and hs-CRP was not performed, and we therefore do not claim independence of the two exposures from each other. In the reverse MR analysis, there was no significant evidence of a genetically proxied reverse association of MDD liability with the TyG index or hs-CRP (**Table 1**). These MR estimates derive from adult or mixed-age GWAS and reflect genetically proxied liability across the life course; they should not be interpreted as effects specific to adolescent-onset depression.

**Table 1:** Bidirectional Mendelian randomization estimates between metabolic-inflammatory traits and major depressive disorder.

Direction	Exposure	Outcome	No. of SNPs	MR Method	OR (95% CI)	P-value
Forward	TyG index	MDD	45	IVW	1.08 (1.03–1.14)	0.001
				MR-Egger	1.06 (1.00–1.12)	0.045
				Weighted median	1.07 (1.02–1.13)	0.005
				MR-PRESSO	1.08 (1.03–1.13)	0.002
Forward	hs-CRP	MDD	52	IVW	1.11 (1.05–1.16)	<0.001
				MR-Egger	1.09 (1.02–1.16)	0.011
				Weighted median	1.10 (1.04–1.16)	0.001
				MR-PRESSO	1.10 (1.05–1.15)	<0.001
Reverse	MDD	TyG index	88	IVW	1.01 (0.98–1.04)	0.45
Reverse	MDD	hs-CRP	88	IVW	1.02 (0.99–1.05)	0.21

ORs are scaled per 1-SD higher genetically predicted exposure. The two primary forward IVW hypotheses were evaluated at a Bonferroni-adjusted threshold of P<0.025; other estimates are reported with nominal P values. Heterogeneity (Cochran’s Q: TyG→MDD, P = 0.18; hs-CRP→MDD, P = 0.09) and MR-Egger intercepts (TyG→MDD, −0.002, P = 0.61; hs-CRP→MDD, 0.001, P = 0.72) suggested no strong heterogeneity or directional pleiotropy; full diagnostics and per-SNP data are provided in **Supplementary Data File S1**. IVW, inverse-variance weighted; MR-PRESSO, mendelian randomization pleiotropy residual sum and outlier; MDD, major depressive disorder; TyG, triglyceride-glucose; hs-CRP, high-sensitivity C-reactive protein; OR, odds ratio; CI, confidence interval.

**Figure 3:**
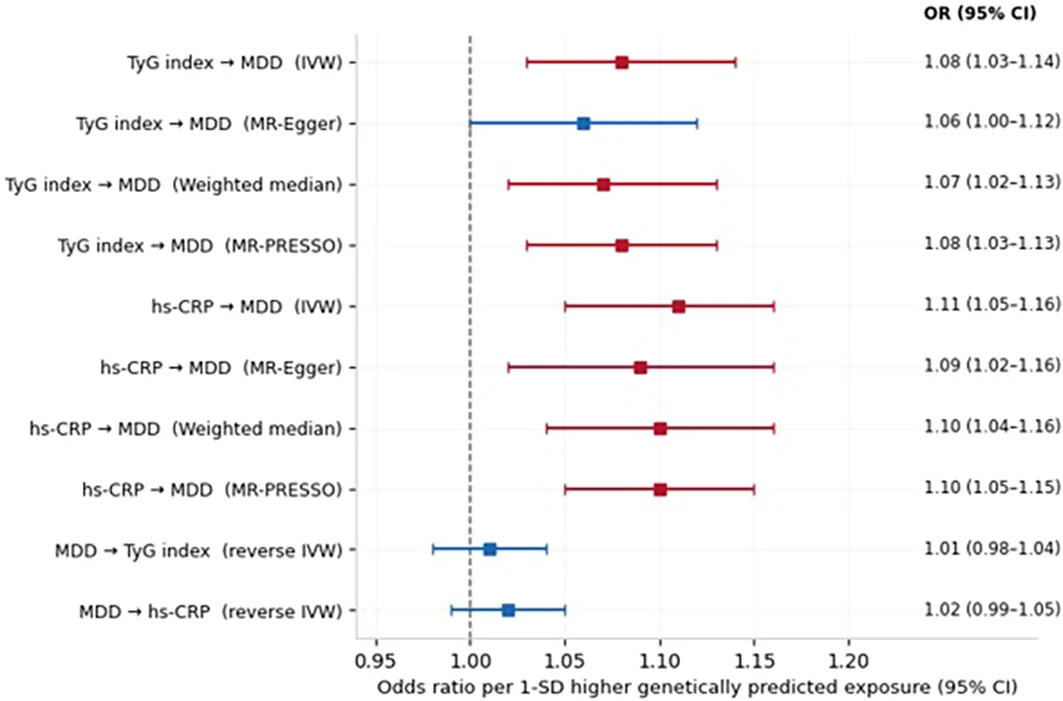
Mendelian randomization estimates for the association of genetically predicted TyG index and hs-CRP with major depressive disorder (MDD), and reverse-direction estimates. IVW, inverse-variance weighted; OR, odds ratio; CI, confidence interval.

### Associations in the late-adolescent NHANES sample

The baseline characteristics of the 327 late adolescents (aged 18–19 years) included in the NHANES analysis are presented in **Table 2**, with a detailed breakdown in **Supplementary Table S3**; counts are raw numbers and percentages are survey-weighted. Forty-one participants met the PHQ-9 ≥10 criterion, corresponding to a survey-weighted prevalence of moderate-to-severe depressive symptoms of 13.2% (SE 2.1%). Compared with participants without depressive symptoms, those with symptoms were more frequently female (58.5% vs 47.6%, weighted P = 0.18), had lower poverty-income ratios, lower educational attainment, and higher mean BMI (25.1 vs 23.9 kg/m², weighted P = 0.07), TyG index (8.44 vs 8.33, weighted P = 0.15), and median hs-CRP (2.6 vs 1.4 mg/L, weighted P = 0.01). In survey-weighted logistic regression with the continuous primary exposures (**Table 3**), each 1-SD higher TyG index was associated with higher odds of moderate-to-severe depressive symptoms in the fully adjusted model (Model C: adjusted OR 1.28, 95% CI 1.01–1.63, P = 0.045), as was each 1-SD higher log-transformed hs-CRP (Model C: adjusted OR 1.33, 95% CI 1.03–1.71, P = 0.027). Secondary categorical analyses were directionally consistent: the highest TyG tertile (adjusted OR 2.02, 95% CI 1.01–4.05; P for trend=0.046) and hs-CRP >3.0 mg/L versus <1.0 mg/L (adjusted OR 1.96, 95% CI 1.02–3.77) were associated with higher odds of depressive symptoms, although confidence intervals were wide given the modest number of events (n=41). The exploratory TyG × hs-CRP multiplicative interaction was positive but imprecise and not statistically significant (OR 2.15, 95% CI 0.92–5.02; P for interaction=0.076; not significant after Benjamini–Hochberg adjustment), and is reported as a statistical interaction only, without mechanistic interpretation. Sensitivity analyses supported the primary findings (**Supplementary Table S6**): associations were directionally consistent when the continuous PHQ-9 score was modeled (adjusted β per 1-SD TyG 0.34, 95% CI 0.05–0.63; per 1-SD log hs-CRP 0.41, 95% CI 0.12–0.70); including participants with hs-CRP >10 mg/L (n=336) attenuated the log-hs-CRP association (adjusted OR 1.24, 95% CI 0.97–1.59, P = 0.089), indicating some sensitivity to extreme inflammatory values; and estimates were materially unchanged after additional adjustment for educational attainment or survey period. Because of the limited number of outcome events, subgroup analyses were restricted and are reported in **Supplementary Table S6** with cautious interpretation.

**Table 2:** Baseline characteristics of participants in the late-adolescent NHANES analytic sample (N = 327) and the retrospective clinical cohort (N = 424).

Characteristic	NHANES: no depressive symptoms (n=286)	NHANES: depressive symptoms (n=41)	P-value	Clinical: control (n=200)	Clinical: depression (n=224)	P-value
Age, years, mean (SD)	18.5 (0.5)	18.5 (0.5)	0.71	16.1 (1.8)	16.2 (1.9)	0.582
Female sex, n (weighted %)	136 (47.6)	24 (58.5)	0.18	120 (60.0)	134 (59.8)	0.965
Race/ethnicity, n (weighted %)			0.34	NA	NA	–
Non-Hispanic White	152 (53.1)	19 (46.3)				
Non-Hispanic Black	38 (13.3)	7 (17.1)				
Hispanic	64 (22.4)	11 (26.8)				
Non-Hispanic Asian/other	32 (11.2)	4 (9.8)				
Poverty-income ratio, n (weighted %)			0.03	NA	NA	–
<1.3 (low)	61 (21.3)	14 (34.1)				
1.3–3.5 (medium)	128 (44.8)	19 (46.3)				
>3.5 (high)	97 (33.9)	8 (19.5)				
Educational attainment, n (weighted %)			0.04	NA	NA	–
Less than high school	47 (16.4)	11 (26.8)				
High school or above	239 (83.6)	30 (73.2)				
BMI, kg/m², mean (SD)	23.9 (4.6)	25.1 (5.3)	0.07	22.5 (3.9)	24.1 (4.3)	<0.001
TyG index, mean (SD)	8.33 (0.52)	8.44 (0.55)	0.15	8.28 (0.4)	8.75 (0.5)	<0.001
hs-CRP, mg/L, median (IQR)	1.4 (0.7–2.9)	2.6 (1.1–5.3)	0.01	0.9 (0.4–1.5)	2.8 (1.2–4.5)	<0.001

For the NHANES sample, values are raw numbers with survey-weighted percentages or survey-weighted means/medians; P values are from survey-weighted tests. For the clinical cohort, values are raw numbers (%) or mean (SD)/median (IQR), and P values are from t-tests, Mann-Whitney U tests, or chi-square tests as appropriate. BMI, body mass index; TyG, triglyceride-glucose index; hs-CRP, high-sensitivity C-reactive protein; IQR, interquartile range; NA, not available; SD, standard deviation.

**Table 3:** Survey-weighted associations between the TyG index, hs-CRP, and moderate-to-severe depressive symptoms (PHQ-9 ≥10) in the late-adolescent NHANES sample (N = 327).

Exposure	Model A OR (95% CI)	Model B OR (95% CI)	Model C OR (95% CI)
Primary (continuous) analyses			
TyG index, per 1-SD	1.34 (1.05–1.71), P = 0.018	1.30 (1.01–1.68), P = 0.043	1.28 (1.01–1.63), P = 0.045
log hs-CRP, per 1-SD	1.42 (1.12–1.80), P = 0.004	1.38 (1.08–1.76), P = 0.010	1.33 (1.03–1.71), P = 0.027
Secondary (categorical) analyses			
TyG tertile 2 vs 1	1.26 (0.62–2.56)	1.22 (0.59–2.52)	1.19 (0.57–2.48)
TyG tertile 3 vs 1	2.18 (1.11–4.28)	2.09 (1.05–4.16)	2.02 (1.01–4.05)
P for trend	0.021	0.034	0.046
hs-CRP 1.0–3.0 vs <1.0 mg/L	1.44 (0.79–2.63)	1.40 (0.76–2.58)	1.36 (0.73–2.53)
hs-CRP >3.0 vs <1.0 mg/L	2.12 (1.12–4.02)	2.04 (1.06–3.93)	1.96 (1.02–3.77)
Exploratory: TyG × hs-CRP interaction	2.41 (1.05–5.53)	2.28 (0.99–5.25)	2.15 (0.92–5.02); P-interaction=0.076

Model A adjusted for age and sex; Model B additionally for race/ethnicity and poverty-income ratio; Model C additionally for BMI. Analyses used rescaled 5.2-year fasting subsample weights with masked strata and primary sampling units. Primary exposures were analyzed as continuous (per 1-SD); categorical analyses are secondary and the interaction is exploratory. P values are nominal; secondary/exploratory P values were additionally subjected to Benjamini–Hochberg false discovery rate control, after which the interaction was not significant. OR, odds ratio; CI, confidence interval; TyG, triglyceride-glucose; hs-CRP, high-sensitivity C-reactive protein.

Covariate missingness, complete-case selection, and the robustness of the primary associations to missing-data handling were evaluated among the 561 exposure/outcome-complete participants (**Supplementary Table S7**). Missingness was greatest for the poverty-income ratio (112 participants, 20.0%), with lower levels for BMI (59, 10.5%) and educational attainment (58, 10.3%); age and sex were complete, and race/ethnicity was missing for 10 participants (1.8%). Participants retained in the complete-case analysis (N = 327) and those excluded because of missing covariates (n=234) showed no major differences in observed age, sex, survey period, TyG index, or depressive-symptom burden (all standardized mean differences ≤0.10); a modest difference was observed for hs-CRP (SMD 0.11). In multiple-imputation analyses including all 561 participants, the primary associations remained directionally consistent with the complete-case estimates (TyG per 1-SD: OR 1.25, 95% CI 1.04–1.50, P = 0.017; log hs-CRP per 1-SD: OR 1.30, 95% CI 1.07–1.58, P = 0.008), supporting the robustness of the primary findings to alternative handling of missing covariate data.

### Associations in the retrospective clinical cohort

**Table 2** summarizes the key characteristics of our clinical cohort, with a comprehensive comparison provided in **Supplementary Table S4**. The clinical cohort of 224 depressed adolescents demonstrated significantly higher mean levels of the TyG index (8.75 vs. 8.28, P<0.001) and median levels of hs-CRP (2.8 vs. 0.9 mg/L, P<0.001) compared to the 200 non-depressed controls.

We performed a multivariable logistic regression analysis to evaluate factors associated with depression (**Table 4**), with covariates (age, sex, BMI) selected based on prior clinical knowledge and the matching design rather than by univariate screening. Consistent with our matching strategy, demographic factors such as age (β = 0.02, P = 0.735) and sex (β = 0.05, P = 0.812) showed no significant association with depression. Notably, the TyG index remained independently associated with depression case status after adjustment for BMI; for every 1-unit increase in the TyG index, the log-odds of depression increased significantly (β = 0.30), corresponding to 35% higher odds (Adjusted OR: 1.35, 95% CI: 1.15–1.58, P < 0.001). Similarly, higher hs-CRP categories showed progressively higher odds of depression case status; adolescents with hs-CRP >3.0 mg/L had the highest positive regression coefficient (β = 0.94), corresponding to 2.5-fold higher odds compared to the reference group (Adjusted OR: 2.55, 95% CI: 1.60–4.06, P < 0.001). Because index blood sampling preceded psychotropic treatment for the current episode and the 11 participants with remote prior exposure had been off psychotropic medication for at least 12 months, acute treatment-related metabolic effects are unlikely to explain the primary estimates; a sensitivity analysis excluding these 11 participants yielded essentially unchanged estimates (TyG: adjusted OR 1.33, 95% CI 1.12–1.57; hs-CRP >3.0 mg/L: adjusted OR 2.41, 95% CI 1.49–3.89). These results are directionally concordant with a contribution of metabolic-inflammatory dysregulation to depression beyond general adiposity, although their observational design precludes causal interpretation.

**Table 4:** Multivariable logistic regression analysis of factors associated with depression in the clinical cohort (N = 424).

Variable	β coefficient	Adjusted OR (95% CI)	P-value
Demographic adjustment factors			
Age (per 1-year increase)	0.02	1.02 (0.91–1.14)	0.735
Female sex (vs male)	0.05	1.05 (0.72–1.54)	0.812
Metabolic and inflammatory factors			
BMI (kg/m²)	0.08	1.09 (0.98–1.21)	0.115
TyG index (per 1-unit increase)	0.30	1.35 (1.15–1.58)	<0.001
hs-CRP <1.0 mg/L	–	1.00 (reference)	–
hs-CRP 1.0–3.0 mg/L	0.35	1.42 (0.98–2.05)	0.062
hs-CRP >3.0 mg/L	0.94	2.55 (1.60–4.06)	<0.001

Covariates (age, sex, BMI) were selected based on prior clinical knowledge and the matching design and entered simultaneously. Odds ratios indicate higher or lower odds of case status, not changes in absolute risk. OR, odds ratio; CI, confidence interval; BMI, body mass index; TyG, triglyceride-glucose index; hs-CRP, high-sensitivity C-reactive protein.

## Discussion

In this comprehensive three-part study, we integrated genetic, population, and clinical evidence to investigate the role of metabolic-inflammatory dysregulation in adolescent and late-adolescent depression. The three components contribute different and complementary levels of evidence. The MR analysis provides genetically proxied evidence that higher TyG index and hs-CRP are associated with higher MDD liability under MR assumptions, with modest effect sizes (ORs of 1.08 and 1.11) and no significant genetically proxied reverse association; because the underlying GWAS were conducted in adult or mixed-age populations, these estimates reflect liability across the life course rather than adolescent-specific effects. The NHANES analysis, now correctly restricted to 327 late adolescents aged 18–19 years, provides population-based observational corroboration: higher TyG index and log hs-CRP were associated with higher odds of moderate-to-severe depressive symptoms in survey-weighted models, with wider confidence intervals appropriate to the sample size. The clinical cohort provides adolescent-specific clinical corroboration, with marked differences in TyG and hs-CRP between depressed adolescents and controls. The directionally concordant observational evidence provides triangulation across distinct study designs but does not independently establish causality (29).

The MR findings provide genetically informed evidence under MR assumptions, but their modest effect sizes warrant measured interpretation: statistical significance at ORs of 1.08–1.11 does not translate directly into clinically meaningful predictive or therapeutic effects. The positive genetically proxied association of the TyG index with depression aligns with literature linking metabolic dysfunction and depressive disorders. Meta-analytic evidence supports associations between type 2 diabetes and depression and between metabolic syndrome and subsequent depressive symptoms (46, 47). Our genetic analysis adds a life-course liability perspective, while adolescent relevance is provided by the observational arms. The biological mechanisms are likely multifactorial. Insulin receptors are widely distributed throughout the brain, and impaired insulin signaling can disrupt crucial neural functions, including synaptic plasticity, neurotransmitter regulation (particularly dopamine and serotonin), and neurogenesis (48, 49). Furthermore, insulin resistance can exacerbate HPA axis dysregulation, leading to chronically elevated cortisol levels, a well-established biological correlate of depression (50). Importantly, our multivariable MR analysis showed that the genetically proxied association of the TyG index with MDD persisted after adjustment for BMI, suggesting that insulin resistance itself, rather than overall adiposity alone, may contribute; we emphasize that this analysis addresses independence from BMI only and does not establish independence of TyG and hs-CRP from each other. More recent MR work has also reported bidirectional genetic associations between type 2 diabetes and depression (51). The BMI-adjusted estimate suggests that general adiposity does not fully account for the genetic association, but the clinical relevance of this distinction in non-obese adolescents requires direct study.

Similarly, the genetically proxied association of hs-CRP with MDD is consistent with the “inflammatory hypothesis of depression” (8, 52). This theory posits that peripheral inflammation can translate into central neuroinflammation, thereby driving depressive pathophysiology. Numerous observational studies have reported elevated inflammatory markers in depressed individuals (53, 54). Pro-inflammatory cytokines can alter the metabolism of tryptophan, shunting it away from serotonin synthesis towards the production of neurotoxic metabolites like quinolinic acid (55). They can also reduce the availability of brain-derived neurotrophic factor (BDNF), impairing neuronal survival and function, and directly impact glial cell function, leading to an altered brain microenvironment conducive to depressive symptoms (8, 56). The null reverse-MR estimates did not support a genetically proxied reverse association; however, they cannot exclude reverse processes operating through non-genetic pathways.

Interpretation of the observational components requires attention to the distinct depression phenotypes used across the three study arms (**Supplementary Table S5**). The MR outcome was GWAS-defined MDD from the PGC meta-analysis, combining diagnostic and broad phenotypes; the NHANES outcome was current moderate-to-severe depressive symptoms over the preceding two weeks (PHQ-9 ≥10), a self-reported screening measure rather than a clinical diagnosis; and the clinical cohort outcome was a psychiatrist-assigned ICD-10 diagnosis (F32.x/F33.x), which may additionally select for treatment-seeking individuals. These phenotypes are related but not interchangeable, and phenotype ascertainment can influence both effect sizes and genetic architecture; we therefore interpret their directional concordance as triangulation across related phenotypes rather than replication of an identical outcome (29, 34).

Ancestry and population context cut both ways. The MR analyses relied on predominantly European-ancestry GWAS; the NHANES sample is drawn from the multi-ancestry US population; and our clinical cohort was recruited at a single Chinese center. The directionally consistent findings across these settings may strengthen interpretation through triangulation across different bias structures (29). However, differences in ancestry, social environment, health systems, and phenotype measurement also limit how broadly the results can be generalized. All three data sources are drawn from high-income or upper-middle-income settings, and our conclusions therefore apply most directly to late adolescents in the US population and to adolescents in comparable clinical settings; replication in larger and more geographically and ancestrally diverse adolescent cohorts is required before broader claims can be made.

Several limitations must be acknowledged. First, the MR analyses relied on GWAS summary statistics from adult or mixed-age, predominantly European-ancestry populations; the resulting estimates reflect genetically proxied liability across the life course and may not transfer directly to adolescence, a period in which hormonal, metabolic, inflammatory, and social contexts differ. Moreover, the TyG source GWAS used random/non-fasting UK Biobank biochemistry, whereas NHANES and clinical TyG were calculated from fasting samples, which may reduce direct cross-component comparability. Second, because PGC MDD2 included a UK Biobank cohort, sample overlap with UK Biobank–based exposure GWAS cannot be excluded or precisely quantified from the summary files used here; stronger instruments reduce, but do not eliminate, overlap-related bias (35). Third, while MR is robust against many forms of confounding, horizontal pleiotropy cannot be entirely ruled out, although our sensitivity analyses detected no strong evidence of it. Fourth, the NHANES analysis was cross-sectional and, despite survey weighting, based on an analytic sample of 327 participants derived from more than 2,000 age-eligible records; we report each exclusion step (**Figure 2**), and reverse causation cannot be excluded. Missing covariate data reduced the complete-case sample from 561 exposure/outcome-complete participants to 327 and could have introduced selection bias if missingness depended on unobserved characteristics. We therefore quantified covariate-specific missingness, compared participants retained in versus excluded from the complete-case sample using standardized mean differences, and performed multiple-imputation sensitivity analyses (**Supplementary Table S7**). The imputed-data estimates were directionally consistent with the complete-case results, reducing, but not eliminating, concern regarding bias from complete-case selection; multiple imputation relies on a missing-at-random assumption conditional on the observed variables included in the imputation model, and residual bias from data missing not at random cannot be excluded. Fifth, excluding participants with hs-CRP >10 mg/L reduces contamination by acute-phase responses (42) but may also remove some participants with genuinely high chronic inflammation, potentially affecting generalizability; our sensitivity analysis including these participants showed attenuation of the hs-CRP association. Sixth, the clinical cohort was retrospective and single-center; although index blood sampling preceded psychotropic treatment for the current episode and the 11 previously exposed participants had been medication-free for at least 12 months, residual confounding related to prior medication exposure, illness severity, or unmeasured socioeconomic factors (unavailable in our EMR) remains possible. Seventh, the MR effect sizes were small and should not be interpreted as evidence of predictive or therapeutic utility. Finally, depression phenotypes differed across the three components, as discussed above. Prospective longitudinal and interventional studies are needed to establish temporal ordering and to test whether modifying metabolic or inflammatory pathways affects depression onset in adolescents.

Despite these limitations, the triangulation of genetic, population-based, and clinical evidence is a methodological strength. The identification of a positive—but imprecise and exploratory—statistical interaction between TyG and hs-CRP in the NHANES sample should be regarded as hypothesis-generating; it reflects a statistical interaction on the odds scale rather than established mechanistic synergy, and it was not a primary conclusion of this study. Recent work emphasizing heterogeneity in energy-homeostasis pathways in depression further argues against treating a combined metabolic-inflammatory signal as a uniform biological subtype (57). Whether the combined burden of insulin resistance and inflammation confers odds beyond their individual associations requires confirmation in larger adolescent samples with adequate statistical power. The observed associations raise the possibility that the TyG index and hs-CRP could be evaluated in future risk-stratification research, but the present study did not assess predictive performance and does not support changes to clinical protocols.

## Conclusion

This triangulation study provides converging but non-equivalent evidence linking metabolic-inflammatory dysregulation with depression in adolescence and late adolescence. Mendelian randomization analyses identified modest genetically proxied associations of the TyG index and hs-CRP with MDD liability across the life course, with the TyG association independent of genetically proxied BMI; observational analyses in late-adolescent NHANES participants and in an adolescent clinical cohort were directionally concordant, with the exploratory TyG × hs-CRP statistical interaction requiring confirmation. Given the reliance of the genetic analyses on adult, predominantly European-ancestry GWAS, the modest effect sizes, and the observational nature of the corroborating data, these findings should be viewed as generating a rationale for prospective risk-prediction studies, mechanistic research, and future intervention trials—rather than as direct support for clinical or preventive strategies—and require replication in larger, more geographically and ancestrally diverse adolescent cohorts.

## Data Availability

The publicly available NHANES datasets and documentation can be accessed through the National Center for Health Statistics (https://wwwn.cdc.gov/nchs/nhanes/). The GWAS summary-statistics resources and their identifiers are described in **Supplementary Table S1**. De-identified data from the retrospective clinical cohort may be made available by the corresponding author upon reasonable request, subject to institutional ethics, privacy, and data-sharing requirements. The analysis code may also be requested from the corresponding author.
