# Supplementary material for: Metabolic and inflammatory traits and depression: a Mendelian randomization study with late-adolescent population-based and clinical triangulation
Source: Front Psychiatry. 2026 Oct 16;17:1935185. doi: 10.3389/fpsyt.2026.1935185 (PMC13720539; doi:10.3389/fpsyt.2026.1935185)
Supplement: Supplementary file 1 [file Table2.docx]

**Supplementary Table S0. Study design, data sources, and participant selection process.**

| **Study phase** | **Data source** | **Time period** | **Population / inclusion** | **Key exclusions** | **Final sample** |
| --- | --- | --- | --- | --- | --- |
| Phase 1: Genetic inference (MR) | UK Biobank–based TyG GWAS^31^; UK Biobank biomarker GWAS (hs-CRP)^32^; GIANT+UK Biobank (BMI)^33^; PGC MDD2 (MDD)^34^ | — | SNPs associated with exposures at P<5×10−8; F-statistic >10; ancestry predominantly European (adult/mixed-age) | LD r²>0.001; palindromic SNPs with MAF>0.42; no proxy SNPs used | IVs: TyG 45 SNPs; hs-CRP 52 SNPs; MDD (reverse) 88 SNPs |
| Phase 2: Population-based corroboration | NHANES 2015–2016 + 2017–March 2020 pre-pandemic (public-use files) | 2015–March 2020 | Late adolescents aged 18–19 y (public PHQ-9 available only for ≥18 y^36^); complete PHQ-9, fasting TG/glucose, hs-CRP, covariates | hs-CRP >10 mg/L (primary analysis); incomplete data (see Figure 2) | N = 327 (depressive symptoms n = 41) |
| Phase 3: Clinical corroboration | Hospital EMR | July 2023–July 2025 | Cases: 12–19 y, ICD-10 F32.x/F33.x; controls: frequency-matched by age/sex | hs-CRP >10 mg/L; autoimmune disease; type 1 diabetes; systemic corticosteroids/antibiotics/lipid-lowering drugs | N = 424 (224 cases, 200 controls) |

EMR, electronic medical records; GWAS, genome-wide association study; IV, instrumental variable; LD, linkage disequilibrium; MAF, minor allele frequency; MDD, major depressive disorder; MR, Mendelian randomization; NHANES, National Health and Nutrition Examination Survey; PHQ-9, Patient Health Questionnaire-9; TG, triglycerides. For Phase 2, covariate-specific missingness, included-versus-excluded comparisons, and multiple-imputation sensitivity analyses are reported in Supplementary Table S7.

**Supplementary Table S1. GWAS data sources used in the Mendelian randomization analyses.**

| **Trait (role)** | **Consortium / database** | **Original publication** | **Sample size** | **Ancestry / age** | **Phenotype definition** | **Genome build** | **Dataset identifier** |
| --- | --- | --- | --- | --- | --- | --- | --- |
| TyG index (exposure) | UK Biobank | Si et al., 2021^31^ | 273,368 | European; 40–69 y | ln[TG (mg/dL) × random/non-fasting glucose (mg/dL)/2]; participants free of diabetes and disorders of lipoprotein metabolism | GRCh37 | UK Biobank Application 51470; Return 3794 (no separate GWAS accession reported) |
| hs-CRP (exposure) | UK Biobank biomarker panel | Sinnott-Armstrong et al., 2021^32^ | 363,228 | Predominantly European; adult | Serum C-reactive protein biomarker GWAS | GRCh37 | UK Biobank field 30710; NIH Figshare DOI 10.35092/yhjc.12355382 |
| BMI (MVMR covariate) | GIANT + UK Biobank | Yengo et al., 2018^33^ | 681,275 | European; adult | Body mass index (kg/m²) | GRCh37 | IEU OpenGWAS ieu-b-40 (PMID 30124842) |
| MDD (outcome / reverse exposure) | Psychiatric Genomics Consortium (MDD2) | Wray et al., 2018^34^ | 135,458 cases / 344,901 controls | European; adult/mixed-age | MDD/major depression case status combining structured interviews, clinician/record-based diagnoses, and broader self-report/help-seeking phenotypes across cohorts | GRCh37 | IEU OpenGWAS ieu-a-1187 (PGC MDD2 public mirror; PMID 29700475) |

Potential sample overlap: PGC MDD2 included a UK Biobank cohort; therefore overlap with the UK Biobank–based TyG and hs-CRP resources (and with UK Biobank in the BMI MVMR source) is possible and could not be precisely quantified from the summary files used here. Strong instruments reduce, but do not eliminate, overlap-related bias^35^. Dataset identifiers denote the most specific publicly traceable resource associated with each source. For the TyG study, no separate GWAS accession was reported, so the UK Biobank Application/Return identifiers are provided. No FinnGen summary statistics were used in the present MR analyses; in Sinnott-Armstrong et al., FinnGen served as an external cohort for downstream polygenic-risk-score evaluation rather than as the source of the biomarker GWAS used here. EHR, electronic health records; MDD, major depressive disorder; MVMR, multivariable Mendelian randomization; TG, triglycerides.

**Supplementary Table S2. Aggregate instrument-strength metrics for the MR analyses.**

| **Exposure** | **No. of SNPs** | **Mean F-statistic** | **Variance explained (R²)** | **GWAS source** |
| --- | --- | --- | --- | --- |
| TyG index | 45 | 419.2 | 6.9% | UK Biobank^31^ |
| hs-CRP | 52 | 581.5 | 8.4% | UK Biobank biomarkers^32^ |

R² and per-SNP F-statistics were recalculated from the GWAS summary statistics as 2 × EAF × (1 − EAF) × β²; the values in the previous version of this table were corrected. The full SNP-level instrument list (SNP, effect/other allele, beta, SE, EAF, exposure P, R², F, outcome beta/SE, harmonization status, palindromic/proxy status) is provided in Supplementary Data File S1. EAF, effect allele frequency; SE, standard error.

**Supplementary Table S3. Detailed baseline characteristics of the late-adolescent NHANES analytic sample (N=327), by depressive-symptom status (PHQ-9 ≥10).**

| **Characteristic** | **Total (N=327)** | **No depressive symptoms (n=286)** | **Depressive symptoms (n=41)** | **P-value** |
| --- | --- | --- | --- | --- |
| Age, years, mean (SD) | 18.5 (0.5) | 18.5 (0.5) | 18.5 (0.5) | 0.71 |
| Female, n (weighted %) | 160 (48.9) | 136 (47.6) | 24 (58.5) | 0.18 |
| PIR <1.3, n (weighted %) | 75 (22.9) | 61 (21.3) | 14 (34.1) | 0.03 |
| Less than high school, n (weighted %) | 58 (17.7) | 47 (16.4) | 11 (26.8) | 0.04 |
| BMI, kg/m², mean (SD) | 24.0 (4.7) | 23.9 (4.6) | 25.1 (5.3) | 0.07 |
| TyG index, mean (SD) | 8.34 (0.52) | 8.33 (0.52) | 8.44 (0.55) | 0.15 |
| hs-CRP, mg/L, median (IQR) | 1.5 (0.7–3.1) | 1.4 (0.7–2.9) | 2.6 (1.1–5.3) | 0.01 |
| Survey period 2015–2016, n (weighted %) | 119 (36.4) | 106 (37.1) | 13 (31.7) | 0.42 |

Raw numbers with survey-weighted percentages; survey-weighted means/medians. PIR, poverty-income ratio; other abbreviations as in Table 2.

**Supplementary Table S4. Detailed baseline characteristics of the retrospective clinical cohort (N=424).**

| **Characteristic** | **Control (n=200)** | **Depression (n=224)** | **P-value** |
| --- | --- | --- | --- |
| Age, years, mean (SD) | 16.1 (1.8) | 16.2 (1.9) | 0.582 |
| Female, n (%) | 120 (60.0) | 134 (59.8) | 0.965 |
| BMI, kg/m², mean (SD) | 22.5 (3.9) | 24.1 (4.3) | <0.001 |
| TyG index, mean (SD) | 8.28 (0.4) | 8.75 (0.5) | <0.001 |
| hs-CRP, mg/L, median (IQR) | 0.9 (0.4–1.5) | 2.8 (1.2–4.5) | <0.001 |
| NLR, mean (SD) | 1.8 (0.6) | 2.2 (0.8) | 0.004 |
| LMR, mean (SD) | 4.5 (1.2) | 3.8 (1.1) | 0.008 |

NLR and LMR were recorded as exploratory inflammatory markers only; they were not primary exposures and did not enter the primary analyses. They are retained here for completeness and moved from the main-text tables to avoid implying a primary analytical role. NLR, neutrophil-to-lymphocyte ratio; LMR, lymphocyte-to-monocyte ratio; other abbreviations as in Table 2.

**Supplementary Table S5. Depression phenotype definitions across the three study components.**

| **Component** | **Phenotype** | **Ascertainment** | **Interpretation** |
| --- | --- | --- | --- |
| MR (PGC MDD2^34^) | Major depressive disorder | GWAS case definitions combining structured interviews, clinician diagnoses, EHR/self-report phenotypes | Lifetime liability to MDD; adult/mixed-age samples |
| NHANES (N=327) | Moderate-to-severe depressive symptoms | PHQ-9 ≥10, self-report, preceding 2 weeks, CAPI administration^37,38^ | Current symptom screening; not a clinical diagnosis |
| Clinical cohort (N=424) | MDD (clinical depression) | Psychiatrist-assigned ICD-10 F32.x/F33.x | Formal clinical ascertainment; possible treatment-seeking selection |

Phenotypes are related but not interchangeable; concordant directions of association are interpreted as triangulation rather than replication of an identical outcome^29^. CAPI, computer-assisted personal interviewing; EHR, electronic health records.

**Supplementary Table S6. Sensitivity and restricted subgroup analyses in the NHANES analytic sample.**

| **Analysis** | **Estimate (95% CI)** | **P-value** |
| --- | --- | --- |
| Continuous PHQ-9 outcome: TyG per 1-SD (Model C) | β 0.34 (0.05–0.63) | 0.022 |
| Continuous PHQ-9 outcome: log hs-CRP per 1-SD (Model C) | β 0.41 (0.12–0.70) | 0.006 |
| Including hs-CRP >10 mg/L (n=336): log hs-CRP per 1-SD (Model C) | OR 1.24 (0.97–1.59) | 0.089 |
| Model C + educational attainment: TyG per 1-SD | OR 1.26 (0.99–1.60) | 0.058 |
| Model C + educational attainment: log hs-CRP per 1-SD | OR 1.30 (1.01–1.68) | 0.041 |
| Model C + survey period indicator: TyG per 1-SD | OR 1.27 (1.00–1.62) | 0.048 |
| Model C + survey period indicator: log hs-CRP per 1-SD | OR 1.32 (1.02–1.70) | 0.031 |

Survey-weighted estimates. Model C adjusts for age, sex, race/ethnicity, poverty-income ratio, and BMI. Given 41 outcome events, subgroup analyses by sex and BMI were restricted and are not emphasized; age-stratified analysis (18 vs 19 years) was not pursued because of limited power.

**Supplementary Table S7. Covariate missingness, complete-case selection, and multiple-imputation sensitivity analyses in the NHANES sample.**

**Panel A. Covariate-specific missingness among the 561 exposure/outcome-complete participants.**

| **Covariate** | **Missing n** | **Missing %** |
| --- | --- | --- |
| Age | 0 | 0.0 |
| Sex | 0 | 0.0 |
| Race/ethnicity | 10 | 1.8 |
| Poverty-income ratio (PIR) | 112 | 20.0 |
| BMI | 59 | 10.5 |
| Educational attainment | 58 | 10.3 |
| Missing ≥1 covariate | 234 | 41.7 |
| Complete-case primary analytic sample | 327 | 58.3 |

Categories are not mutually exclusive; for example, 5 participants were missing both PIR and BMI. The “missing ≥1 covariate” count (234) corresponds to the final exclusion step in Figure 2 (561 − 234 = 327). Percentages are of 561. PIR, poverty-income ratio; BMI, body mass index.

**Panel B. Observed characteristics of participants retained in versus excluded from the complete-case sample.**

| **Characteristic** | **Complete cases (N=327)** | **Excluded (n=234)** | **SMD** |
| --- | --- | --- | --- |
| Age, years, mean (SD) | 18.5 (0.5) | 18.5 (0.5) | 0.02 |
| Female, n (weighted %) | 160 (48.9) | 120 (51.3) | 0.05 |
| Survey period 2015–2016, n (weighted %) | 119 (36.4) | 88 (37.6) | 0.03 |
| TyG index, mean (SD) | 8.34 (0.52) | 8.37 (0.55) | 0.06 |
| hs-CRP, mg/L, median (IQR) | 1.5 (0.7–3.1) | 1.8 (0.8–3.4) | 0.11 |
| PHQ-9 score, mean (SD) | 3.4 (3.8) | 3.8 (4.1) | 0.10 |
| PHQ-9 ≥10, n (weighted %) | 41 (13.2) | 34 (14.8) | 0.05 |

Raw numbers with survey-weighted percentages; survey-weighted means/medians. Standardized mean differences (SMDs) were computed on observed data (log-transformed hs-CRP); variables with missing values (PIR, BMI, educational attainment) are not compared between groups because they are unobserved in the excluded group. hs-CRP, high-sensitivity C-reactive protein; PHQ-9, Patient Health Questionnaire-9.

**Panel C. Complete-case versus multiple-imputation estimates for the primary exposures (Model C).**

| **Exposure** | **Complete-case (N=327), OR (95% CI)** | **Multiple imputation (N=561, m=50), OR (95% CI)** | **Interpretation** |
| --- | --- | --- | --- |
| TyG index, per 1-SD | 1.28 (1.01–1.63), P=0.045 | 1.25 (1.04–1.50), P=0.017 | Directionally consistent; similar magnitude |
| log hs-CRP, per 1-SD | 1.33 (1.03–1.71), P=0.027 | 1.30 (1.07–1.58), P=0.008 | Directionally consistent; similar magnitude |

Complete-case estimates correspond to Table 3 (Model C). Multiple imputation by chained equations with 50 imputed datasets; only covariates were imputed (predictive mean matching for PIR and BMI, logistic regression for educational attainment, polytomous regression for race/ethnicity); TyG, hs-CRP, PHQ-9 outcomes, survey weights, strata, and primary sampling units were not imputed. Survey-weighted logistic models were fitted within each imputed dataset using the rescaled 5.2-year fasting subsample weights, and estimates were combined using Rubin’s rules. OR, odds ratio; CI, confidence interval.
