# Supplementary material for: Metabolic and inflammatory traits and depression: a Mendelian randomization study with late-adolescent population-based and clinical triangulation
Source: Front Psychiatry. 2026 Oct 16;17:1935185. doi: 10.3389/fpsyt.2026.1935185 (PMC13720539; doi:10.3389/fpsyt.2026.1935185)
Supplement: Supplementary file 2 [file Table3.docx]

STROBE-MR Checklist of Recommended Items to Address in Reports of Mendelian Randomization Studies

**Completed for:** Metabolic and Inflammatory Traits and Depression: A Mendelian Randomization Study With Late-Adolescent Population-Based and Clinical Triangulation

**Manuscript version: Revised manuscript — Round 2 (NHANES primary complete-case N = 327)**

| **Item No.** | **Section** | **Checklist item** | **Page No.** | **Relevant text from manuscript** |
| --- | --- | --- | --- | --- |
| **TITLE AND ABSTRACT** | | | | |
| 1 | Title and abstract | Indicate Mendelian randomization (MR) as the study’s design in the title and/or the abstract if that is a main purpose of the study. | pp. 1–2 | The title explicitly identifies a “Mendelian Randomization Study.” The Abstract Methods states that bidirectional two-sample MR was performed using GWAS summary statistics, with IVW as the primary method plus sensitivity and multivariable MR analyses. |
| **INTRODUCTION** | | | | |
| 2 | Background | Explain the scientific background and rationale for the reported study. What is the exposure? Is a potential causal relationship between exposure and outcome plausible? Justify why MR is a helpful method to address the study question. | pp. 3–5 | The Introduction describes insulin resistance (TyG index) and systemic inflammation (hs-CRP) as biologically plausible contributors to depression, summarizes confounding and reverse-causation concerns in observational studies, and explains why genetically informed MR can help address these limitations. |
| 3 | Objectives | State specific objectives clearly, including pre-specified causal hypotheses (if any). State that MR is a method that, under specific assumptions, intends to estimate causal effects. | p. 5; p. 13 | The final Introduction paragraph states the primary aim: to use two-sample MR to estimate genetically proxied associations of TyG index and hs-CRP with MDD, interpreted as life-course genetic liability rather than adolescent-specific effects. The Statistical Analysis section explicitly states that IVW estimates the genetically proxied association “under MR assumptions.” No claim of pre-registration is made. |
| **METHODS** | | | | |
| 4 | Study design and data sources | Present key elements of the study design early in the article. Consider including a table listing sources of data for all phases of the study. For each data source contributing to the analysis, describe the following: | p. 6; Supplementary Table S0, p. 1 | A three-stage triangulation design is described early in Methods: bidirectional two-sample MR, cross-sectional NHANES corroboration, and a retrospective clinical case-control study. Supplementary Table S0 summarizes the data sources, periods, inclusion/exclusion criteria, and final samples. |
| 4a | Setting | Setting: Describe the study design and the underlying population, if possible. Describe the setting, locations, and relevant dates, including periods of recruitment, exposure, follow-up, and data collection, when available. | pp. 7–11; Supplementary Table S0, p. 1 | MR sources are described as predominantly adult/mixed-age European-ancestry GWAS (UK Biobank, GIANT + UK Biobank, PGC MDD2). NHANES includes pooled 2015–2016 and 2017–March 2020 pre-pandemic cycles, restricted to ages 18–19. The clinical cohort was recruited from the hospital EMR between July 2023 and July 2025. |
| 4b | Participants | Participants: Give the eligibility criteria, and the sources and methods of selection of participants. Report the sample size, and whether any power or sample size calculations were carried out prior to the main analysis. | pp. 7–12; Figure 2, pp. 10–11; Supplementary Tables S0–S1, pp. 1–3 | GWAS sample sizes and ancestry/age characteristics are provided for TyG (n=273,368), hs-CRP (n=363,228), BMI (n≈681,275), and PGC MDD2 (135,458 cases/344,901 controls). NHANES selection is shown stepwise to final N=327. Clinical cohort eligibility is reported for 224 MDD cases and 200 frequency-matched controls. No prospective MR sample-size calculation was performed because the analysis used fixed, publicly available GWAS summary statistics; instrument strength was evaluated using per-SNP and aggregate F-statistics and R². |
| 4c | Genetic variants | Describe measurement, quality control and selection of genetic variants. | pp. 11–12; Supplementary Tables S1–S2, pp. 2–3; Supplementary Data File S1 | Instrument selection used P<5×10⁻⁸, LD clumping r²<0.001 within 10,000 kb, F-statistic >10, exclusion of variants absent after harmonization, no proxy SNPs, and exclusion of palindromic SNPs with MAF>0.42. Per-SNP R²/F-statistics and harmonization status are stated to be provided in Supplementary Data File S1. |
| 4d | Variables | For each exposure, outcome, and other relevant variables, describe methods of assessment and diagnostic criteria for diseases. | pp. 7–12; Supplementary Table S1, pp. 2–3; Supplementary Table S5, pp. 4–5 | TyG, hs-CRP, BMI, and MDD GWAS sources and phenotype definitions are reported. NHANES PHQ-9 scoring/administration, fasting glucose/triglycerides, hs-CRP assays, PIR, and education are described. Clinical MDD is psychiatrist-assigned ICD-10 F32.x/F33.x; blood sampling and hs-CRP assay details are provided. Supplementary Table S5 contrasts the three depression phenotypes. |
| 4e | Ethics | Provide details of ethics committee approval and participant informed consent, if relevant. | p. 7; p. 26 | The clinical cohort was exempted from formal ethical review by the institutional IRB (GZPH-IRB-2024-001). NHANES and GWAS datasets had ethics approval/informed consent from the original investigators; reuse of public data required no new approval. |
| 5 | Assumptions | Explicitly state the three core IV assumptions for the main analysis (relevance, independence and exclusion restriction) as well assumptions for any additional or sensitivity analysis. | p. 7 | The Methods now explicitly state the three core instrumental-variable assumptions: (1) relevance, whereby the genetic variants are robustly associated with the exposure; (2) independence, whereby the variants are independent of confounders of the exposure–outcome association; and (3) exclusion restriction, whereby the variants influence the outcome only through the exposure. Relevance is operationalized through genome-wide significance and F-statistic thresholds, while potential violations of independence/exclusion restriction are considered through pleiotropy, heterogeneity, sensitivity analyses, and the Discussion of limitations. |
| 6 | Statistical methods: main analysis | Describe statistical methods and statistics used. | pp. 12–14 | Random-effects IVW is the primary MR estimator, supplemented by MR-Egger, weighted median, MR-PRESSO, and MVMR. Forward and reverse MR are performed. Bonferroni correction is used for the two primary forward IVW hypotheses. |
| 6a | Quantitative variables | Describe how quantitative variables were handled in the analyses (i.e., scale, units, model). | pp. 9, 12–14; Table 1, p. 16 | MR effects are reported per 1-SD higher genetically predicted exposure. TyG and hs-CRP scales/definitions are described, and ORs with 95% CIs are used for MDD. NHANES continuous TyG and log hs-CRP are standardized per 1-SD for primary observational models. |
| 6b | Genetic variants / weights | Describe how genetic variants were handled in the analyses and, if applicable, how their weights were selected. | pp. 11–13 | Independent instruments were selected by LD clumping, harmonized across exposure/outcome datasets, variants unavailable in the outcome data were excluded, proxy SNPs were not used, and ambiguous palindromic SNPs were excluded. IVW, weighted-median, MR-Egger and MR-PRESSO estimators are described. |
| 6c | MR estimator and covariates | Describe the MR estimator (e.g. two-stage least squares, Wald ratio) and related statistics. Detail the included covariates and, in case of two-sample MR, whether the same covariate set was used for adjustment in the two samples. | pp. 12–14; Supplementary Table S1, pp. 2–3 | The primary estimator is random-effects IVW; MR-Egger, weighted median and MR-PRESSO are sensitivity estimators. MVMR adjusts the TyG association for genetically proxied BMI. The manuscript identifies the underlying GWAS sources, but source-GWAS covariate-adjustment sets are not described in detail or explicitly compared between exposure and outcome samples. |
| 6d | Missing data | Explain how missing data were addressed. | pp. 8, 11–14; Figure 2, pp. 10–11; Supplementary Table S7, pp. 6–7 | For MR harmonization, variants unavailable in the outcome dataset were excluded and no proxy SNPs were used. For NHANES, participants with incomplete PHQ-9, fasting triglyceride/glucose, hs-CRP, or required covariate data were excluded, with stepwise counts shown in Figure 2. In this round, the primary NHANES analysis retained the fixed complete-case cohort (N=327); covariate-specific missingness among the 561 exposure/outcome-complete participants, an included-versus-excluded comparison using standardized mean differences, and a multiple-imputation sensitivity analysis (m=50, chained equations, covariates only; Rubin’s rules) were added to evaluate missing-data robustness (Supplementary Table S7). |
| 6e | Multiple testing | If applicable, indicate how multiple testing was addressed. | p. 13; Table 1 footnote, p. 16 | The two primary forward IVW hypotheses (TyG→MDD and hs-CRP→MDD) were evaluated using a Bonferroni-adjusted threshold of P<0.025. Reverse MR and sensitivity estimators were reported with nominal P values. |
| 7 | Assessment of assumptions | Describe any methods or prior knowledge used to assess the assumptions or justify their validity. | pp. 11–16; Table 1, p. 16; Supplementary Table S2, p. 3 | Instrument strength was assessed with F-statistics and R². Horizontal pleiotropy/heterogeneity were evaluated using MR-Egger intercepts, Cochran’s Q, and MR-PRESSO. LD clumping and allele harmonization procedures are described. |
| 8 | Sensitivity analyses and additional analyses | Describe any sensitivity analyses or additional analyses performed (e.g. comparison of effect estimates from different approaches, independent replication, bias analytic techniques, validation of instruments, simulations). | pp. 13–20; Supplementary Tables S6–S7, pp. 5–7 | MR-Egger, weighted median, and MR-PRESSO were used to test robustness; MVMR adjusted TyG for genetically proxied BMI; bidirectional MR assessed the reverse direction. NHANES sensitivity analyses included continuous PHQ-9, inclusion of hs-CRP >10 mg/L, education/survey-period adjustment, and in Round 2 a multiple-imputation analysis of missing covariates in all 561 exposure/outcome-complete participants. Clinical medication-exclusion sensitivity analyses were also performed. Observational analyses are interpreted as triangulating rather than validating evidence. |
| 9a | Software | Name statistical software and package(s), including version and settings used. | pp. 12–14 | Analyses used R version 4.3.1. MR packages/functions are named: TwoSampleMR (harmonise_data, mr, mr_heterogeneity, mr_pleiotropy_test) and MRPRESSO (mr_presso). Specific package version numbers are not reported. |
| 9b | Pre-registration | State whether the study protocol and details were pre-registered (as well as when and where). | Not preregistered | The study protocol and MR analyses were not prospectively preregistered; therefore, no registry identifier is available. This is reported transparently here rather than presented as an unreported item. |
| **RESULTS** | | | | |
| 10a | Participant flow | Report the numbers of individuals at each stage of included studies and reasons for exclusion. Consider use of a flow diagram. | pp. 7–11; Figure 2, pp. 10–11; Supplementary Table S0, p. 1 | GWAS source sample sizes are reported. NHANES participant selection is shown in a flow diagram from 2,150 age-eligible participants to final N=327, with reasons for each exclusion. Clinical cohort final numbers are 224 cases and 200 controls. |
| 10b | Descriptive data | Report summary statistics for phenotypic exposure(s), outcome(s), and other relevant variables (e.g. means, SDs, proportions). | pp. 17–20; Tables 2–4, pp. 18–20; Supplementary Tables S3–S5, pp. 3–5 | The observational components report demographic, TyG, hs-CRP and depression/depressive-symptom summary statistics. MR source sample sizes/phenotype definitions are summarized in Supplementary Table S1. |
| 10c | Meta-analysis heterogeneity | If the data sources include meta-analyses of previous studies, provide the assessments of heterogeneity across these studies. | Source GWAS publication (Wray et al., 2018; Ref. 34) | PGC MDD2 is a published meta-analysis of contributing cohorts. Cohort-level heterogeneity pertains to the original source GWAS and was not re-estimated from the summary statistics used in the present study; readers are referred to Wray et al. (2018) for source-study meta-analytic details. Heterogeneity across genetic instruments in the present MR analyses is reported separately using Cochran’s Q. |
| 10d(i) | Two-sample MR: sample similarity | Provide justification of the similarity of the genetic variant-exposure associations between the exposure and outcome samples. | pp. 7–8, 23; Supplementary Table S1, pp. 2–3 | The manuscript documents that source GWAS are predominantly European ancestry but differ in age/population composition, and it explicitly treats those differences as a limitation rather than assuming full comparability. |
| 10d(ii) | Two-sample MR: sample overlap | Provide information on the number of individuals who overlap between the exposure and outcome studies. | pp. 8, 23; Supplementary Table S1, pp. 2–3 | PGC MDD2 included a UK Biobank cohort, so overlap with UK Biobank-based TyG/hs-CRP exposure GWAS is possible. The exact number of overlapping participants could not be quantified from the summary files; this limitation is explicitly stated. |
| 11a | Variant associations | Report the associations between genetic variant and exposure, and between genetic variant and outcome, preferably on an interpretable scale. | Supplementary Data File S1 (separate); Supplementary Table S2, p. 3 | The manuscript states that Supplementary Data File S1 contains SNP, effect/other alleles, exposure beta/SE/P, EAF, R², F-statistic, outcome beta/SE, and harmonization status for each instrument. Aggregate instrument strength is reported in Supplementary Table S2. |
| 11b | MR estimates | Report MR estimates of the relationship between exposure and outcome, and the measures of uncertainty from the MR analysis, on an interpretable scale, such as odds ratio or relative risk per SD difference. | pp. 15–17; Table 1, p. 16; Figure 3, p. 16 | Forward and reverse MR estimates are reported as ORs with 95% CIs and P values per 1-SD higher genetically predicted exposure. Primary IVW estimates: TyG OR 1.08 (95% CI 1.03–1.14) and hs-CRP OR 1.11 (95% CI 1.05–1.16). |
| 11c | Absolute risk | If relevant, consider translating estimates of relative risk into absolute risk for a meaningful time period. | Not applicable | The MR analysis estimates odds of MDD liability using summary-level GWAS data and does not provide a meaningful absolute-risk time horizon; the manuscript therefore does not translate MR ORs into absolute risk. |
| 11d | Plots | Consider plots to visualize results (e.g. forest plot, scatterplot of associations between genetic variants and outcome versus between genetic variants and exposure). | Figure 3, p. 16 | A forest plot displays IVW, MR-Egger, weighted-median, MR-PRESSO, and reverse-direction MR estimates with 95% CIs. |
| 12a | Validity of assumptions | Report the assessment of the validity of the assumptions. | pp. 15–17; Supplementary Table S2, p. 3 | Instrument strength is reported (mean F-statistics 419.2 for TyG and 581.5 for hs-CRP; R² 6.9% and 8.4%). MR-Egger intercepts, Cochran’s Q, and MR-PRESSO results are reported; no strong evidence of directional pleiotropy or significant MR-PRESSO outliers was detected. |
| 12b | Additional statistics | Report any additional statistics (e.g., assessments of heterogeneity across genetic variants, such as I², Q statistic or E-value). | pp. 15–17; Table 1, p. 16; Supplementary Table S2, p. 3 | Cochran’s Q P values are reported (TyG→MDD P=0.18; hs-CRP→MDD P=0.09), together with MR-Egger intercepts and instrument F-statistics/R². |
| 13a | Sensitivity to assumption violations | Report any sensitivity analyses to assess the robustness of the main results to violations of the assumptions. | pp. 15–17; Table 1, p. 16 | Effect directions are compared across IVW, MR-Egger, weighted median and MR-PRESSO; pleiotropy and heterogeneity diagnostics are reported. |
| 13b | Other additional analyses | Report results from other sensitivity analyses or additional analyses. | pp. 15–20; Supplementary Tables S6–S7, pp. 5–7 | MVMR adjusting for genetically proxied BMI is reported (TyG OR 1.07, 95% CI 1.01–1.13). Observational sensitivity analyses and clinical medication-exclusion sensitivity analysis are also reported as triangulating evidence. |
| 13c | Direction of causal relationship | Report any assessment of direction of causal relationship (e.g., bidirectional MR). | pp. 11–17; Table 1, p. 16; Figure 3, p. 16 | Bidirectional MR includes TyG→MDD, hs-CRP→MDD, MDD→TyG, and MDD→hs-CRP. Reverse IVW estimates were not statistically significant. |
| 13d | Comparison with non-MR analyses | When relevant, report and compare with estimates from non-MR analyses. | pp. 17–24; Tables 2–4, pp. 18–20 | NHANES and clinical observational results are presented separately and compared directionally with the MR results in the Discussion. The manuscript explicitly frames this as triangulation across non-equivalent evidence rather than mutual validation. |
| 13e | Additional plots | Consider additional plots to visualize results (e.g., leave-one-out analyses). | Figure 3, p. 16; no leave-one-out plot reported | A main forest plot is provided. Leave-one-out and SNP-level scatter/funnel plots were not prespecified as primary reporting outputs; robustness is instead evaluated with MR-Egger, weighted median, MR-PRESSO, Cochran’s Q, and per-SNP instrument information in Supplementary Data File S1. |
| **DISCUSSION** | | | | |
| 14 | Key results | Summarize key results with reference to study objectives. | pp. 21–24 | The Discussion summarizes MR, NHANES, and clinical findings, emphasizes modest MR effect sizes and directionally concordant observational evidence, and returns to the triangulation objective. |
| 15 | Limitations | Discuss limitations of the study, taking into account the validity of the IV assumptions, other sources of potential bias, and imprecision. Discuss both direction and magnitude of any potential bias and any efforts to address them. | pp. 23–24 | Limitations include adult/mixed-age European GWAS, non-fasting TyG source GWAS versus fasting observational samples, possible UK Biobank sample overlap, residual horizontal pleiotropy, NHANES selection/reverse causation, hs-CRP >10 mg/L exclusion, retrospective single-center clinical design, residual medication/socioeconomic confounding, small MR effects, and phenotype heterogeneity. |
| 16a | Interpretation: meaning | Meaning: Give a cautious overall interpretation of results in the context of their limitations and in comparison with other studies. | pp. 21–25 | The manuscript uses cautious “genetically proxied association” language, distinguishes statistical from clinical importance, compares findings with previous metabolic/depression and inflammation/depression literature, and avoids claiming observational validation of causality. |
| 16b | Interpretation: mechanism | Mechanism: Discuss underlying biological mechanisms that could drive a potential causal relationship between the investigated exposure and the outcome, and whether the gene-environment equivalence assumption is reasonable. Use causal language carefully, clarifying that IV estimates may provide causal effects only under certain assumptions. | pp. 21–23 | Potential mechanisms discussed include brain insulin signaling, synaptic plasticity, neurotransmitter regulation, HPA-axis effects, peripheral/central inflammation, tryptophan metabolism, BDNF, and glial function. Causal wording is explicitly calibrated to MR assumptions. The manuscript also addresses the practical implication of gene–environment equivalence by emphasizing that adult/mixed-age MR estimates reflect genetically proxied life-course liability and should not be assumed to represent the effects of short-term pharmacologic, behavioral, or adolescent-onset modification of TyG or hs-CRP. |
| 16c | Interpretation: clinical relevance | Clinical relevance: Discuss whether the results have clinical or public policy relevance, and to what extent they inform effect sizes of possible interventions. | pp. 24–25 | The manuscript states that the observed associations may motivate future risk-stratification research but that predictive performance was not assessed and the findings do not support changes to clinical protocols or immediate clinical/preventive strategies. |
| 17 | Generalizability | Discuss the generalizability of the study results (a) to other populations, (b) across other exposure periods/timings, and (c) across other levels of exposure. | pp. 23–25 | Generalizability is discussed across predominantly European MR data, multi-ancestry US NHANES data, and a single-center Chinese cohort. Adult/mixed-age versus adolescent timing and non-fasting versus fasting exposure ascertainment are acknowledged. Replication in larger, geographically and ancestrally diverse adolescent cohorts is recommended. |
| **OTHER INFORMATION** | | | | |
| 18 | Funding | Describe sources of funding and the role of funders in the present study and, if applicable, sources of funding for the databases and original study or studies on which the present study is based. | p. 27 | The manuscript states: “No specific funding was received for this study.” Funding and funder roles for the underlying GWAS and NHANES source studies are reported in their respective original publications and official data documentation; those source-study funding statements were not reproduced in full in the present article. |
| 19 | Data and data sharing | Provide the data used to perform all analyses or report where and how the data can be accessed, and reference these sources in the article. Provide the statistical code needed to reproduce the results in the article, or report whether the code is publicly accessible and if so, where. | p. 26; Supplementary Table S1, pp. 2–3 | Dataset/resource identifiers are given for TyG, hs-CRP, BMI and PGC MDD2; NHANES public access is identified. De-identified clinical data are available on reasonable request subject to privacy/ethics. Analysis code is to be deposited in OSF/Zenodo upon acceptance or is available on reasonable request. |
| 20 | Conflicts of interest | All authors should declare all potential conflicts of interest. | p. 26 | The authors declare no commercial or financial relationships that could be construed as a potential conflict of interest. |

**Checklist source and attribution:** STROBE-MR (Strengthening the Reporting of Observational Studies in Epidemiology using Mendelian Randomization), Skrivankova VW et al., JAMA. 2021;326(16):1614–1621, and the STROBE-MR Explanation and Elaboration, BMJ. 2021;375:n2233. Official checklist available from strobe-mr.org. The checklist is distributed under CC BY 3.0.
